# Childhood Trauma and *COMT* Genotype Interact to Increase Hippocampal Activation in Resilient Individuals

**DOI:** 10.3389/fpsyt.2016.00156

**Published:** 2016-09-14

**Authors:** Sanne J. H. van Rooij, Jennifer S. Stevens, Timothy D. Ely, Negar Fani, Alicia K. Smith, Kimberly A. Kerley, Adriana Lori, Kerry J. Ressler, Tanja Jovanovic

**Affiliations:** ^1^Department of Psychiatry and Behavioral Sciences, Emory University School of Medicine, Atlanta, GA, USA; ^2^McLean Hospital, Department of Psychiatry, Harvard Medical School, Belmont, MA, USA

**Keywords:** *COMT* Val158Met polymorphism, depression, functional magnetic resonance imaging, hippocampus, posttraumatic stress disorder, response inhibition, resilience

## Abstract

Both childhood trauma and a functional catechol-O-methyltransferase (*COMT*) genetic polymorphism have been associated with posttraumatic stress disorder (PTSD) and depression; however, it is still unclear whether the two interact and how this interaction relates to long-term risk or resilience. Imaging and genotype data were collected on 73 highly traumatized women. DNA extracted from saliva was used to determine *COMT* genotype (Val/Val, *n* = 38, Met carriers, *n* = 35). Functional MRI data were collected during a Go/NoGo task to investigate the neurocircuitry underlying response inhibition. Self-report measures of adult and childhood trauma exposure, PTSD and depression symptom severity, and resilience were collected. Childhood trauma was found to interact with *COMT* genotype to impact inhibition-related hippocampal activation. In Met carriers, more childhood trauma was associated with decreased hippocampal activation, whereas in the Val/Val group childhood trauma was related to increased hippocampal activation. Second, hippocampal activation correlated negatively with PTSD and depression symptoms and positively with trait resilience. Moreover, hippocampal activation mediated the relationship between childhood trauma and psychiatric risk or resilience in the Val/Val, but not in the Met carrier group. These data reveal a potential mechanism by which childhood trauma and *COMT* genotype interact to increase risk for trauma-related psychopathology or resilience. Hippocampal recruitment during inhibition may improve the ability to use contextual information to guide behavior, thereby enhancing resilience in trauma-exposed individuals. This finding may contribute to early identification of individuals at risk and suggests a mechanism that can be targeted in future studies aiming to prevent or limit negative outcomes.

## Introduction

Trauma exposure during childhood is a well-known risk factor for later development of psychiatric disorders, such as posttraumatic stress disorder (PTSD) and depression (1). However, not everyone who experiences childhood trauma develops a psychiatric disorder; in fact, some do quite well and become high-functioning resilient individuals (2), which is potentially linked to resilience traits or processes. Genetics are one potential component that may contribute to resilient traits. A better understanding of mechanisms for psychiatric risk or resilience after childhood trauma exposure is needed for the identification of individuals at risk and delivery of early interventions.

Differential responses to stressful life events such as childhood trauma may be explained in part by genetic risk factors. One of the more common genes studied in mental illness is the Val158Met substitution of the catechol-O-methyltransferase (*COMT*) gene. The enzyme COMT regulates dopamine (DA) levels in the brain, and its activity depends on the *COMT* gene, located on chromosome 22q11.2 (3). A functional single nucleotide polymorphism (SNP), rs4680, codes the substitution of valine (Val) by methionine (Met) (3). This substitution is associated with lower enzymatic activity, resulting in higher extracellular DA levels (4). The Met allele has been related to increased risk for PTSD and was found to interact with trauma load (5, 6). The likelihood to develop depression after exposure to adverse life events (7) or during stressful periods (8) is also higher in individuals carrying the Met allele. Furthermore, childhood adversity (e.g., parental loss, divorce, financial problems) interacts with Met allele genotype to increase the risk for depression in adulthood (9).

Although numerous studies have shown associations of *COMT* with PTSD and depression (5–9), the mechanisms underlying this gene–disease association are not clear. It is possible that polymorphisms of this gene affect brain structure, function, or related cognitive processes following exposure to trauma. These neural and cognitive changes may then, in turn, increase the likelihood for developing psychopathology or enhance resilience. A cognitive process that may be affected by both *COMT* polymorphism and childhood trauma is response inhibition. Response inhibition is the ability to suppress a behavior that is no longer required or inappropriate given environmental demands (10). This process appears to be impaired in several psychiatric disorders (11–13) and is thought to be mediated by the prefrontal cortex (PFC) (14). The ventromedial (vm) PFC in particular has been associated with inhibition of both fear and non-emotional responses, and reduced activation was observed in patients with PTSD (15, 16) and depression (17). Another part of the PFC, i.e., the dorsolateral PFC (DLPFC), is implicated in cognitive control and therefore also thought to play a role in response inhibition (18). Because of previous work specifically showing the role of the vmPFC in PTSD (15, 16), here we used the vmPFC as a PFC region of interest (ROI). The hippocampus plays an essential role in contextual learning and memory and is therefore thought to be involved in regulation of inhibitory processes based on contextual information (19). Hippocampal volume has consistently been shown to be smaller in both PTSD (20) and depression (21). Hippocampal function is also found to be impaired in PTSD (12, 16, 22); however, the literature on inhibition-related hippocampal function in depression is limited. *COMT* may impact inhibition-related functioning. DA, regulated by COMT, is thought to be an important neuromodulator of inhibitory processes (23), and *COMT* is primarily expressed in the PFC and hippocampus (24). A previous study indeed showed impaired inhibition and safety learning in Met/Met carriers. The relationship was specifically found among individuals who met criteria for PTSD (25).

Response inhibition develops well into adolescence and early adulthood (26), and childhood trauma may, therefore, influence the development of inhibition-related processes and brain regions that support inhibition. Indeed, childhood trauma has been associated with reduced volume of hippocampal (27–30) and prefrontal regions (30–32). Increases in inhibition-related activity in a part of the vmPFC, the rostral anterior cingulate cortex (ACC), have been observed in maltreated participants compared to healthy controls (33, 34), and maltreated youth with PTSD symptoms have shown decreased response inhibition-related vmPFC activation (33). We recently observed an association between childhood trauma and decreased inhibition-related rACC activation in adults with PTSD, but not in a traumatized control group (35). This evidence suggests that childhood trauma impacts inhibition-related brain processes, however, not in all individuals exposed to early trauma, pointing to a potential risk mechanism for childhood trauma-related psychopathology. However, this has not been investigated to date.

In the current functional magnetic resonance imaging (fMRI) study, a response inhibition task was used to examine the inhibition-related neurocircuitry in traumatized women from inner-city Atlanta with varying levels of childhood trauma exposure and a range of PTSD and depression symptoms. Even though the group that was studied was specifically at increased risk for PTSD, they were also at greater risk for depression, given the high comorbidity between the two disorders (36), therefore, depression scores were also assessed. We hypothesized that the *COMT* polymorphism may interact with childhood trauma to influence response inhibition-related brain functioning. More specifically, we hypothesized that more childhood trauma is associated with decreased inhibition-related activation in the vmPFC and hippocampus only in the risk (Met carrier) group and with more vmPFC and hippocampal activation in the Val/Val group. Second, we expect that hippocampal and vmPFC activation correlate negatively with PTSD and depression symptoms and positively with trait resilience. Finally, we hypothesize that the relationship between childhood trauma and psychiatric risk or resilience is mediated by hippocampal and vmPFC activation. As particularly volumetric measures of the hippocampus showed differences for left and right in PTSD (20), we analyzed the left and right hippocampus separately.

## Materials and Methods

### Participants

A total of 116 African-American women aged 18–62 years were recruited through the Grady Trauma Project, an ongoing study of risk factors for PTSD. Participants were approached in the waiting rooms of primary care medical clinics of a publicly funded hospital that serves a low-income minority population in inner-city Atlanta. Participants were invited for a MRI scan if they had experienced at least one criterion A trauma according to the DSM-IV and if they did not meet one of the exclusion criteria: medical or physical conditions that preclude MRI scanning (e.g., metal implants), neurological disorder, history of head injury or loss of consciousness (>5 min), history of a psychotic disorder, and current psychotropic medication. Participants had normal or corrected-to-normal vision. Patients were excluded when urine tests for pregnancy or illegal drug use (conducted within 24–48 h of the MRI scan) were positive. After complete written and verbal description of the study, all participants provided written-informed consent. The Institutional Review Board of Emory University and the Research Oversight Committee of Grady Memorial Hospital approved the study procedures. Testing took place at Grady Memorial Hospital and the Biomedical Imaging Technology Center of Emory University. Functional MRI data from a subset of these participants have been reported elsewhere (15).

### Clinical Assessment

Well-validated questionnaires were used to assess trauma exposure during childhood [Childhood Trauma Questionnaire (CTQ)] (37, 38) and adulthood [Traumatic Events Inventory (TEI)] (39). The total score for CTQ (of the 25 recommended items) and TEI were used as continuous measures. The Modified PTSD Symptom Scale (PSS) (40) was used to measure current PTSD symptoms, and the Beck Depression Inventory (BDI) (41) assessed current depression symptoms. Trait-level resilience was assessed using the Connor–Davidson Resilience scale (CD-RISC); a higher score on this measure is indicative of greater psychological resilience (42).

### Response Inhibition Task

Response inhibition was measured using the Go/NoGo task that followed previous work by Leibenluft et al. (43) and is described in more detail elsewhere (15). On all trials, a white X or O appeared on a black screen for 1000 ms. Participants were instructed to respond as fast as possible to this Go signal by pressing a 1 for X and 2 for O. However, when the NoGo signal (i.e., red rectangle behind X or O) appeared, they were instructed to withhold their response. The stimulus event was followed by a jittered inter-trial interval ranging from 1250 to 2500 ms, and a 500-ms white fixation cross. The task consisted of four runs separated by three 20 s rest periods. Each run comprised 26 “Go” trials, 13 “NoGo” trials, and 14 blank trials distributed randomly.

### Genotyping

DNA was extracted from saliva in Oragene collection vials (DNA Genotek Inc., ON, Canada) using the DNAdvance kit (Beckman Coulter Genomics, Danvers, MA, USA) and genotyped as previously reported (25). The *COMT* Val^158^Met SNP, rs4680, was genotyped using the Sequenom iPlex chemistries and the MassARRAY system (Sequenom Inc., San Diego, CA, USA). The assay cell rate was 97.6%. Within and across plate duplicates were used for quality control. All duplicates were concordant. Genotypes for control samples (identified as those without PTSD) were in Hardy–Weinberg Equilibrium (*p* > 0.05). HWE was performed in all genotyped samples and was used to confirm that the genotype is accurate. Participants who were homozygous for the G-allele (Val/Val; *N* = 38) were compared to individuals who carried at least one A-allele (Met/Val or Met/Met; *N* = 35). This “dominant” model has previously been used to address the skewed genotypic distributions.

### Brain Imaging Acquisition and Analyses

Functional and structural images were acquired on a Siemens 3.0-Tesla Magnetom Trio TIM whole-body MR scanner (Siemens, Malvern, PA, USA) using a 12-channel head coil. Functional images were acquired using the Z-SAGA pulse sequence (44) to minimize signal loss due to susceptibility artifacts. Volumes contained 26 axial slices acquired parallel to the anterior–posterior commissure line with TR = 2530 ms, TE 1 = 30 ms, TE 2 = 67 ms, flip angle = 90°, and voxel size 3.75 mm × 3.75 mm × 4 mm. Structural images were acquired using a gradient-echo, T1-weighted pulse sequence (176 slices, TR = 2600 ms, TE = 3.02 ms, 1 mm^3^ voxel size).

Functional data were preprocessed and analyzed with SPM 5^1^ (45). Preprocessing included slice time correction (with a high-pass filter), motion correction, realignment to the first volume in the series, coregistration of the structural image to the mean of the realigned functional images, and spatial normalization to the International Consortium for Brain Mapping (ICBM) 152-subject template using the voxel-based morphometry (VBM) toolbox.^2^ Functional images were smoothed with an 8-mm Gaussian kernel. For each participant, event-related responses were modeled for correct and incorrect trials in the NoGo and Go conditions, convolved with a canonical hemodynamic response function. Only correct trials were included, but mean accuracy was high and did not differ between the PTSD and control groups (see Table 1). To correct for head motion, the six participant-specific realignment parameters were included as regressors of no interest. Inhibition was defined as the linear contrast of the NoGo relative to the Go condition.

**Table 1:** **Clinical and demographic characteristics of the Val/Val and Met carriers group**.

	Val/Val (*N* = 38)		Met carriers (*N* = 35)		*t*-test	*p*-value
	M	SD	M	SD
Age (years)	36.4	12.7	38.6	12.0	*t*_(71)_ = −0.75	0.46
% Correct: NoGo condition	98.9	1.9	98.0	5.0	*t*_(71)_ = 0.99	0.32
% Correct: Go condition	98.4	1.9	97.3	7.3	*t*_(71)_ = 0.87	0.38
Adult trauma load (TEI adult total score)	3.6	1.8	3.9	2.2	*t*_(71)_ = −0.58	0.56
Childhood trauma load (CTQ total score)	39.9	15.7	46.0	18.3	*t*_(71)_ = −1.55	0.13
Depression severity (BDI total score)	12.0	8.9	11.6	8.2	*t*_(71)_ = 0.21	0.83
PTSD symptom severity (PSS total score)	12.9	11.0	14.4	12.3	*t*_(71)_ = −0.55	0.58
Intrusive symptoms	2.9	2.9	3.8	3.8	*t*_(71)_ = −1.19	0.24
Avoidance/numbing symptoms	5.7	5.3	5.4	5.6	*t*_(71)_ = 0.22	0.82
Hyperarousal symptoms	4.3	3.9	5.2	3.8	*t*_(71)_ = −0.95	0.35
Resilience (CD-RISC total score)	33.0	5.4	33.4	6.0	*t*_(60)_ = −0.27	0.80

*No significant differences (*p* < 0.05) between groups*.

*CTQ, childhood trauma questionnaire (37, 38); TEI, traumatic events inventory (39); PSS, Modified PTSD Symptom Scale (40); BDI, Beck Depression Inventory (41); CD-RISC, Connor–Davidson Resilience Scale (42)*.

The T1 scan was processed using FreeSurfer (available at http://surfer.nmr.mgh.harvard.edu), a freely available and extensively validated automated parcellation software program. The FreeSurfer analysis was completed with a single version (5.3.0) and a single OS (Redhat Enterprise Linus 6). Technical details on the FreeSurfer analysis are described elsewhere [e.g., Ref. (46, 47)]. Neuroanatomical labels were automatically assigned to the volumes. This automatic labeling was based on probabilistic information from a manually labeled set and was previously shown to give similar results as when manually labeled (48). A standardized protocol (available at http://enigma.ini.usc.edu/protocols/imaging-protocols/) was used to check the quality of the segmentations before the group analyses were performed. The volumes of the left and right hippocampus and the vmPFC [a combination of the by FreeSurfer defined medial orbitofrontal cortex and lateral orbitofrontal cortex (49)] were used for the group analyses.

### Group Analyses

#### *COMT* and Childhood Trauma Effects

For the fMRI data, ROI analyses were performed for the left and right hippocampus and the vmPFC. The left and right hippocampus ROIs were defined using a structural atlas, i.e., the Automated Anatomical Labeling (AAL) atlas. For the vmPFC, a 6-mm sphere around a peak voxel (MNI coordinates: 4,44,−4) of a recent study showing reduced vmPFC activation (15) was used. Mean contrast estimates for the ROIs were extracted using SPM and were included as dependent variables in moderated regression analyses. *COMT* genotype group, CTQ, and COMT*CTQ were included in three moderated regression analyses to predict inhibition-related left hippocampal, right hippocampal and vmPFC activation, respectively. Age was also included as a predictor to correct for age effects on inhibition. Additional analyses controlling for ancestry and adult trauma load were conducted.

Secondary whole brain analyses were performed to investigate the interaction of *COMT* genotype with childhood trauma, covarying for age and main effects of CTQ and genotype, outside the predefined ROIs. A combined height-extent threshold was used to correct for multiple comparisons. A cluster-forming threshold of *p* < 0.01 was used, and when combined with a cluster size of *k* = 28 resulted in a corrected probability of *p* < 0.05 (voxel-wise probability *p* = 0.00008). This was calculated by implementing Monte Carlo simulation using AlphaSim within the REST toolbox for SPM5 (50) for voxels within a gray-matter mask based on the ICBM 152 atlas, with 1000 iterations.

Finally, structural analyses were performed to investigate main effects of *COMT* genotype, CTQ and COMT*CTQ on regional volume of the hippocampus and vmPFC. The moderated regression analyses were corrected for age and intracranial volume.

#### Correlation Analyses

To investigate the relationship between inhibition-related BOLD activation in the ROIs with psychiatric outcome and resilience, correlation analyses with clinical measures of PTSD (PSS total score) and depression (BDI total score) and the measure for resilience (CD-RISC total) were conducted. Additional analyses were performed with age, CTQ, and ancestry as covariates. Separate analyses were performed to investigate the relationship between subtypes of childhood trauma and inhibition-related activation.

#### Moderated Mediation Analysis

To test the third hypothesis that the relationship between childhood trauma and psychiatric risk or resilience is mediated by inhibition-related hippocampal or vmPFC activation, moderated mediation analyses were performed separately for PTSD symptoms, depression symptoms, and resilience. Childhood trauma was included as the independent variable, PSS, BDI, or CD-RISC as the dependent variables, inhibition-related activation as the mediator, and *COMT* genotype as the moderator.

## Results

### Participants

After excluding for scanner or stimulus presentation issues (*N* = 6), anatomical abnormalities (e.g., falx calcification; *N* = 11), excessive head motion (>3 mm; *N* = 16), and poor task performance (>80% incorrect NoGo trials, *N* = 10), the final sample consisted of 73 participants: 38 Val/Val and 35 Met carriers (34 Val/Met and 1 Met/Met). The two genotype groups did not differ in age, behavioral task performance, adult trauma load, childhood trauma, PTSD or depression symptoms, and resilience (Table 1).

### *COMT* and Childhood Trauma Effects

**Figure 1:**
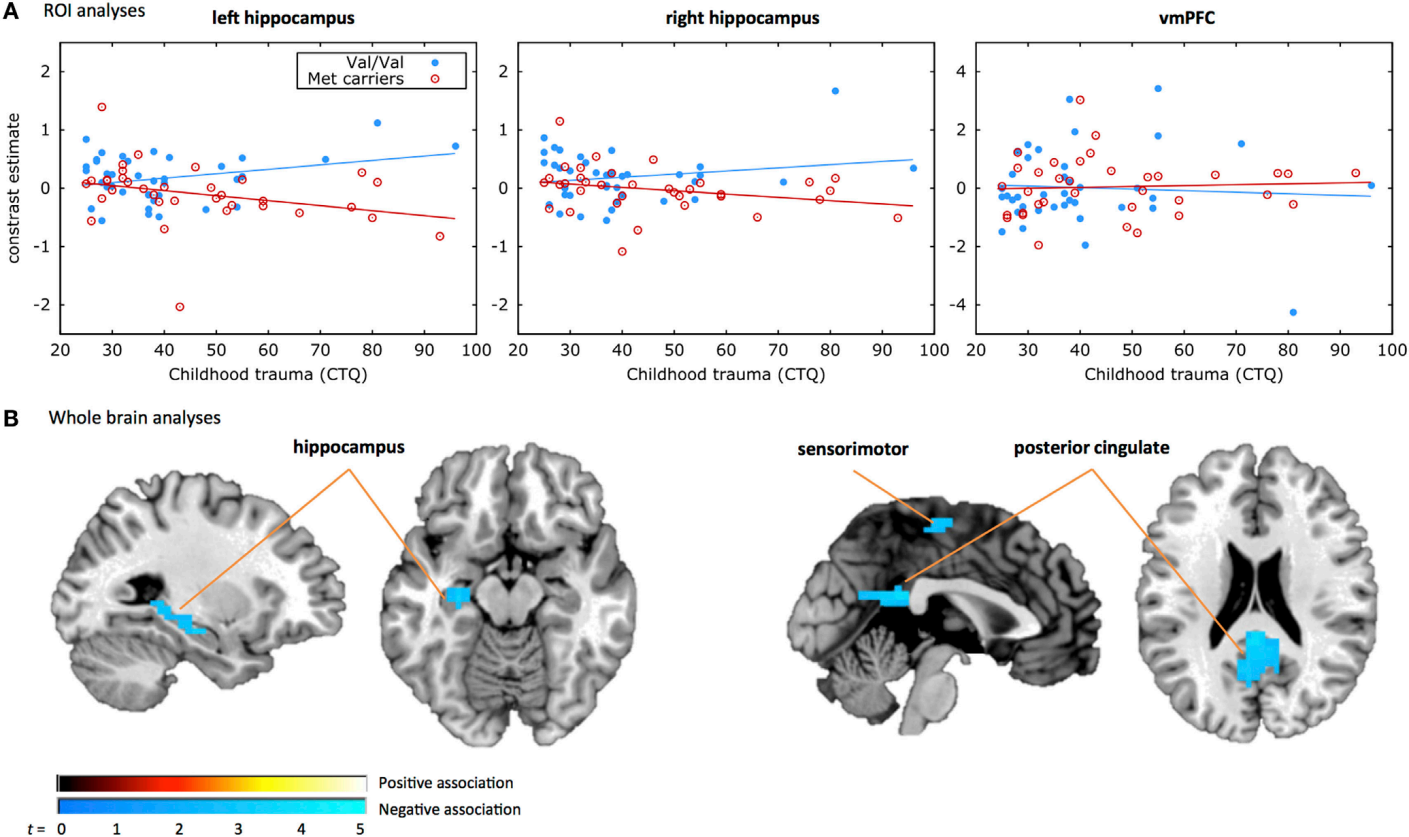
***COMT* by childhood trauma interaction**. Displayed are the results from **(A)** the region of interest (ROI) analyses and **(B)** the whole brain analyses of the *COMT* genotype by childhood trauma interaction. **(A)** Shows the response inhibition-related activation in the three ROIs, i.e., left hippocampus (left), right hippocampus (middle), and ventromedial prefrontal cortex (vmPFC) on the *y*-axis plotted against the CTQ score on the *x*-axis. The filled blue dots represent individuals with the Val/Val genotype and the red open dots represent individuals carrying the Met allele. A significant *COMT* genotype by childhood trauma effect was observed in the left hippocampus, with less inhibition-related hippocampal activation with increasing levels of childhood trauma in the Met carrier group and more inhibition-related hippocampal activation with increasing levels of childhood trauma in the Val/Val group. **(B)** Shows the whole brain activation during the response inhibition task for the *COMT* genotype by childhood trauma effect. The positive association indicates that the Met carrier group shows more inhibition-related activation with increased rates of childhood trauma compared to the Val/Val group. The negative association indicates that the Met carrier group shows less inhibition-related activation with increased rates of childhood trauma compared to the Val/Val group. A significant *COMT* genotype by childhood trauma effect was observed in the hippocampus, sensorimotor, and posterior cingulate areas.

Results of the moderated regression analyses are shown in Table 2 and Figure 1A. The contrast values comparing correct NoGo trials with correct Go trials were extracted for the left and right hippocampus and the vmPFC. The regression model for the left hippocampus was significant [*F*_(4,68)_ = 3.357, *p* = 0.014, *R*^2^ = 0.165, *R*^2^*adjusted* = 0.116], revealing a main effect of *COMT* genotype (*p* = 0.020) and a significant interaction between *COMT* genotype and childhood trauma (*p* = 0.012) on inhibition-related left hippocampal activation. The regression model for the right hippocampus was not significant [*F*_(4,68)_ = 2.268, *p* = 0.071, *R*^2^ = 0.118. *R*^2^*adjusted* = 0.066], and the model for vmPFC was also not significant (*p* > 0.1). Figure 1A shows that more childhood trauma (CTQ total score) was associated with more inhibition-related hippocampal activation in the Val/Val group, but less hippocampal activation in the Met carrier group. The main effect of *COMT* group was explained by more hippocampal activation in the Val/Val group compared to the Met carrier group.

**Table 2:** **Results of moderated regression analyses**.

	Standardized *B*	*t*	*p*-value
**a. Left hippocampus**			
Age	−0.05	−0.48	0.634
CTQ	−0.02	−0.15	0.881
*COMT*	−0.27	−2.39	0.020*
CTQ*COMT	−0.29	−2.60	0.012*
**b. Right hippocampus**			
Age	−0.08	−0.65	0.515
CTQ	−0.004	−0.03	0.974
*COMT*	−0.24	−2.06	0.043
CTQ*COMT	−0.23	−1.98	0.051
**c. vmPFC**			
Age	0.09	0.76	0.451
CTQ	−0.02	−0.15	0.885
*COMT*	0.004	0.03	0.973
CTQ*COMT	0.06	0.52	0.603

*CTQ, childhood trauma questionnaire (37, 38)*.

*Indicates p < 0.05.

The secondary whole brain results of the interaction of *COMT* genotype and childhood trauma are shown in Figure 1B and Table 3. Childhood trauma score interacted with *COMT* genotype to influence activation in the left hippocampus, posterior cingulate, and right sensorimotor areas. Main effects of *COMT* genotype are shown in Table 3 and Table S1 in Supplementary Material. Regarding the structural analyses, there was no significant *COMT* genotype by childhood trauma interaction or main effects of *COMT* genotype or childhood trauma on left or right hippocampal or vmPFC volume.

**Table 3:** **Results of whole brain analyses**.

		MNI coordinates				
Region	HEM	*x*	*y*	*z*	*Z*	*k*
**Interaction between *COMT* genotype and CTQ**						
*Met carriers with increasing CTQ > Val/Val with increasing CTQ*						
No significant clusters						
*Val/Val with increasing CTQ > Met carriers with increasing CTQ*						
Paracentral lobule	R	8	−28	60	3.75	78
Supp. motor area	R	12	−12	60	3.64	
Postcentral G.	R	28	−28	60	3.42	
Posterior cingulate G.	–	0	−44	20	3.58	57
Precuneus	L	−4	−60	20	3	
Cuneus	L	−4	−72	24	2.69	
Hippocampus	L	−28	−20	−16	3.22	34
Parahippocampal G.	L	−32	−40	−4	2.89	
**Effect of *COMT* genotype**						
*Positive correlation with # met alleles*						
Precuneus	R	20	−52	48	4.33	30
Sup. Occipital G.	R	24	−76	40	3.36	31
Cuneus	R	20	−68	32	3.34	
*Negative correlation with # met alleles*						
Parahippocampal G.	L	−24	−28	−16	3.22	28
Hippocampus	L	−24	−16	−16	2.9	
Hippocampus	L	−20	−16	−8	2.86	
Cerebellum-vermis	–	0	−44	−12	2.95	32
Cerebellum-vermis	–	0	−56	−12	2.77	
Cerebellum	R	8	−44	−16	2.74	

### Correlation between Inhibition-Related Activation and Psychiatric Outcome and Resilience

**Figure 2:**
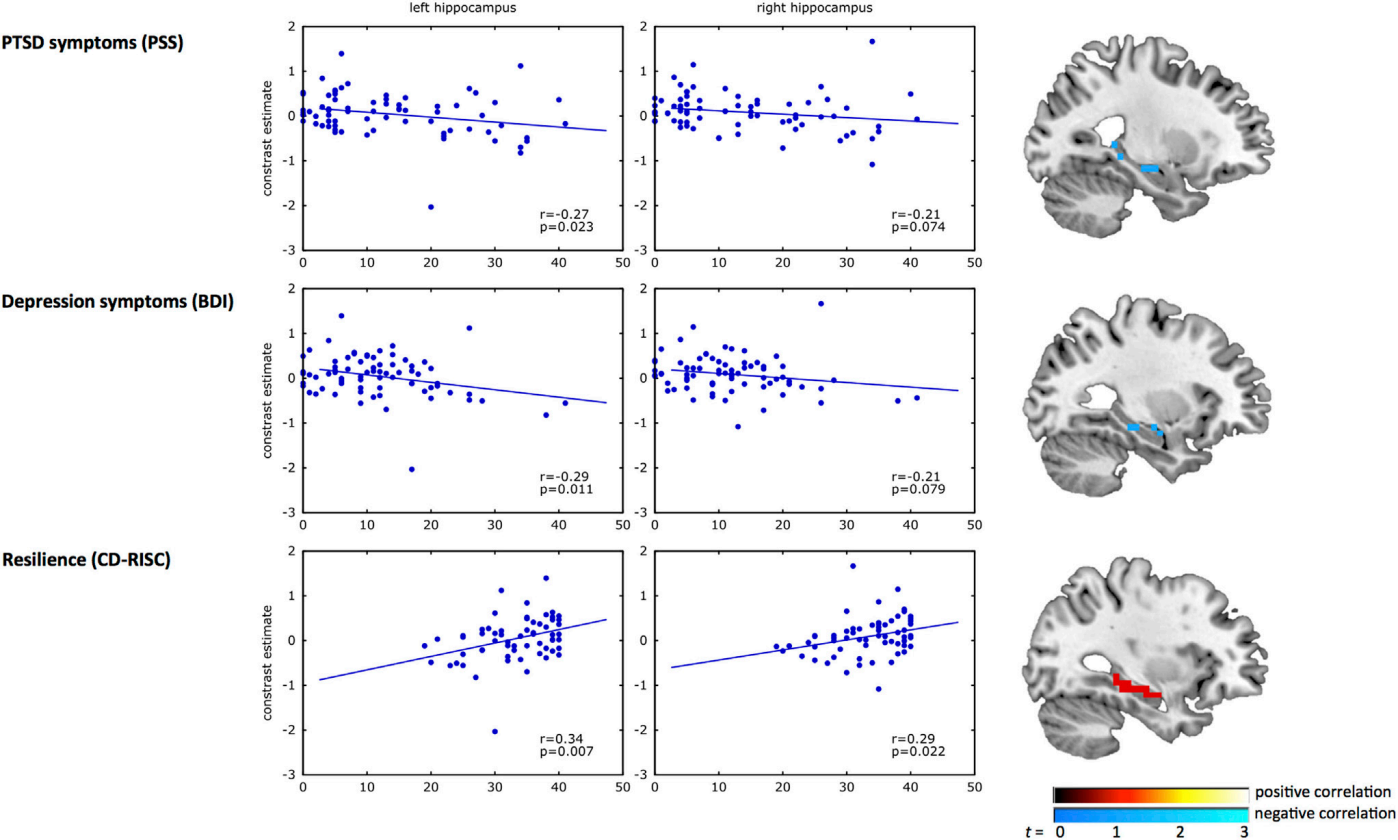
**Correlation analyses of hippocampal activation with psychiatric outcome and resilience**. The scatterplots show the correlation of response inhibition-related BOLD activation in the left and right hippocampus with PTSD symptoms (top panel), depression symptoms (middle panel), and resilience (bottom panel). In each plot, the blue dots represent the inhibition-related activation of each participant (*y*-axis) plotted against the clinical measure for PTSD symptoms [Modified PTSD Symptom Scale (PSS) (40)], depression symptoms [Beck Depression Inventory (BDI) (41)], and resilience [Connor–Davidson Resilience scale (CD-RISC) (42)] on the *x*-axis. The brain images on the right show the uncorrected (*p* < 0.01) activation within the structural hippocampal ROI, presented for illustrative purposes only. A negative correlation was observed between inhibition-related hippocampal activation and PSS and BDI, whereas a positive correlation with CD-RISC was observed.

Results from the correlation analyses between clinical measures and the left and right hippocampus are presented in Figure 2. A significant negative correlation was observed between inhibition-related left hippocampal activation and PTSD (*r* = −0.27, *p* = 0.02) and depression symptoms (*r* = −0.29, *p* = 0.01). Correlations of clinical measures with the right hippocampus did not reach significance (PTSD, *r* = −0.21, *p* = 0.07; depression, *r* = −0.21, *p* = 0.08). In contrast, resilience (CD-RISC score) correlated positively with inhibition-related activation in the left (*r* = 0.34, *p* = 0.007), and right hippocampus (*r* = 0.29, *p* = 0.02).

### Relationship between Subtypes of Childhood Trauma and Inhibition-Related Activation

The *COMT* groups did not differ in total childhood trauma levels, or the subtypes of physical and emotional abuse or neglect; however, the Met carrier group had experienced higher levels of childhood sexual abuse (mean: 11.66, SD: 6.33) than the Val/Val group (mean: 8.11, SD: 4.8; *t*_(71)_ = 2.7, *p* = 0.008).

Correlation analyses were performed to investigate if a particular type of childhood trauma was related to inhibition-related activation in the two *COMT* groups. In the Val/Val group a significant positive correlations was observed between left hippocampal activation and both physical (*r* = 0.35, *p* = 0.030) and sexual abuse (*r* = 0.33, *p* = 0.042). In the Met carrier group, a significant negative correlation was demonstrated between left hippocampal activation and emotional abuse (*r* = −0.38, *p* = 0.026). No significant correlations were observed with the right hippocampus or the vmPFC.

### Controlling for Ancestry and Adult Trauma

#### Sensitivity Analysis Controlling for Population Substructure

To ensure that the association between X and Y is not the result of confounding due to admixture in our all African-American sample, we used the first principal component *via* principal component analysis (PCA) from those subjects with genome-wide data as a covariate to in our X model.

For a subset of our sample (*N* = 70), genome-wide data from the Omni Express BeadChip (Illumina, San Diego, CA, USA) were available for PCA. PLINK (51) was used to perform quality control analyses such that SNPs that had a call rate <98%, a minor allele frequency (MAF) < 0.01, or significant deviation from Hardy–Weinberg proportions were excluded, as were samples with >2% missing data and related samples. A set of roughly autosomal independent markers (~50,000 SNPs) was selected by pruning the data in windows of 50 base pairs, removing one SNP from each pair of SNPs with *r*^2^ > 0.05 (PLINK). PCA was then performed to infer axes of ancestry.

#### Regression and Correlation Analyses Controlled for Ancestry and Adult Trauma

The first principal component (PC1) derived from genome-wide data was added as an additional factor in the moderated regression analyses. The main effect of *COMT* genotype, as well as the interaction between *COMT* and CTQ on left inhibition-related hippocampal activation remained significant after adding PC1 as an additional factor, *F*_(5,64)_ = 2.545 *p* = 0.037, *R*^2^ = 0.166, *R*^2^*adjusted* = 0.101 (Table S1A in Supplementary Material). The model for the right hippocampus did again not reach significance, *F*_(5,64)_ = 1.687, *p* = 0.151, *R*^2^ = 0.116, *R*^2^ *adjusted* = 0.047 (Table S1B in Supplementary Material), nor did the model for the vmPFC, *F*_(5,64)_ = 0.257, *p* = 0.935, *R*^2^ = 0.020, *R*^2^ *adjusted* = −0.057 (Table S1C in Supplementary Material).

Including adult trauma as an additional factor did also not affect these results (Table S2 in Supplementary Material), and, again, a main effect of *COMT* genotype and COMT*CTQ effect on left hippocampal activation was observed, *F*_(6,63)_ = 2.307, *p* = 0.045, *R*^2^ = 0.180, *R*^2^ *adjusted* = 0.102 (Table 2A in Supplementary Material). Again, the model for the right hippocampus, *F*_(6,63)_ = 1.561, *p* = 0.174, *R*^2^ = 0.129, *R*^2^ *adjusted* = 0.046 (Table S2B in Supplementary Material), and vmPFC, *F*_(6,63)_ = 0.387, *p* = 0.885, *R*^2^ = 0.036, *R*^2^ *adjusted* = 0.056 (Table S2C in Supplementary Material), were not significant.

Partial correlation analyses were performed to investigate the separate and combined effect of including age, childhood trauma (CTQ total), and ancestry (PC1) as covariates in the analyses. The significant correlation between inhibition-related left hippocampus activation and PTSD symptoms, depression symptoms, and resilience was not affected by the separate or combined inclusion of the covariates (Table S3 in Supplementary Material). The correlation between right hippocampal activation and PTSD symptoms became significant when only age was included as a covariate, but not with any of the other covariates (Table S3 in Supplementary Material). Furthermore, right hippocampal activation did not correlate with depression symptoms, and this was not affected by including the covariates. Right hippocampal activation correlated significantly with resilience, and this correlation remained significant after covarying for age, CTQ total, and ancestry (Table S3 in Supplementary Material).

### Moderated Mediation Analysis

**Figure 3:**
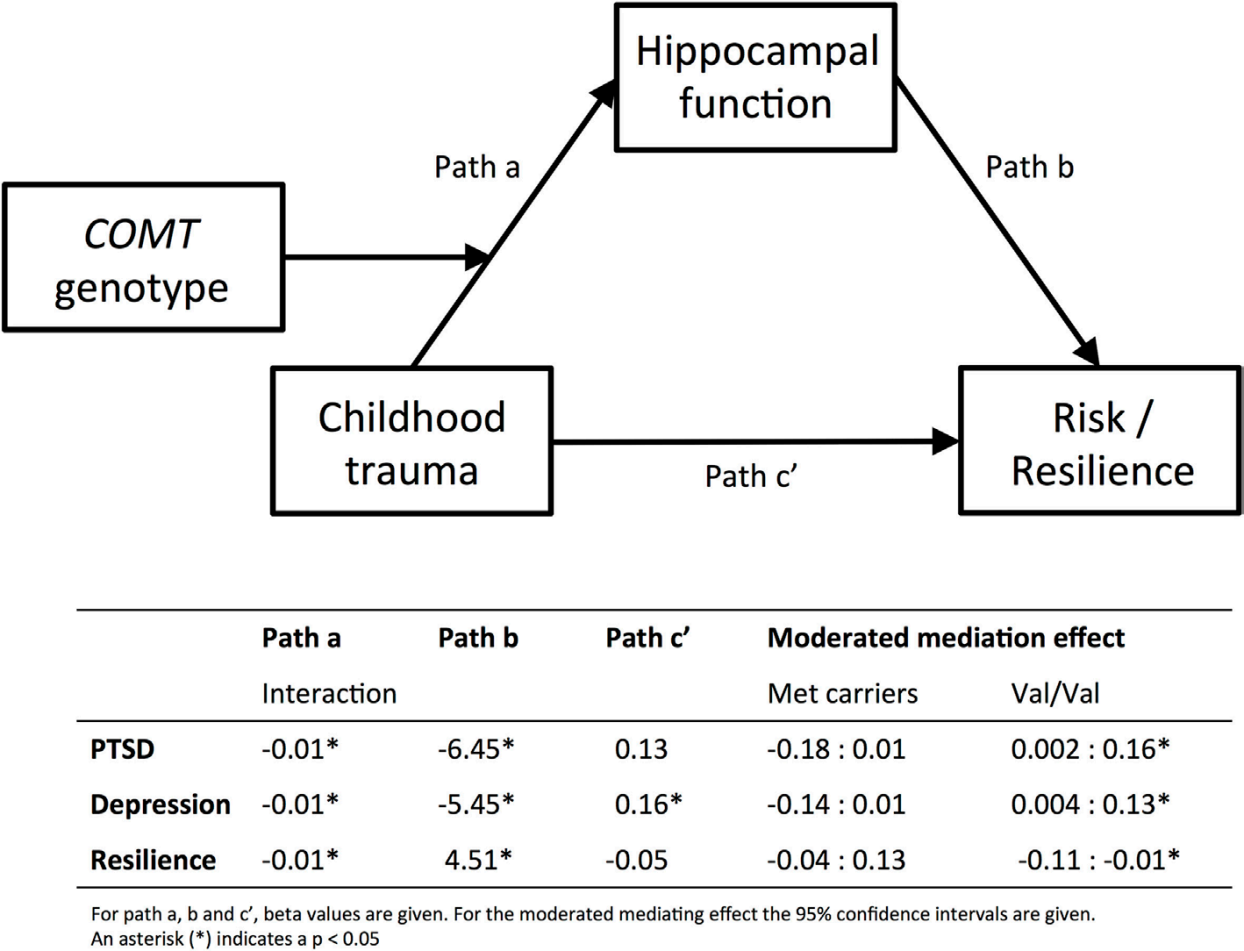
**Moderated mediation analyses**. Results from the moderated mediation analyses are displayed. The top panel shows the model and the bottom part shows the corresponding beta values for path a, b and c′, separately for PTSD, depression, and resilience. Path a corresponds to the results presented in Figure 1, and shows that COMT genotype moderates the effect of childhood trauma [measured with CTQ, Childhood Trauma Questionnaire (37, 38)] on inhibition-related hippocampal activation. Path b corresponds to the results displayed in Figure 2, hippocampal function predicts PTSD [PSS, Modified PTSD Symptom Scale (40)], depression [BDI; Beck Depression Inventory (41)], or resilience [CD-RISC, Connor–Davidson Resilience scale (42)]. For the moderated mediation analysis, the 95% confidence intervals are given in the table. The effect of childhood trauma on psychiatric risk or resilience is mediated by hippocampal function, but only in the Val/Val group. Finally, Path c′ shows that childhood trauma only directly predicts depression symptoms.

Figure 3 shows the results of the moderated mediation analysis for left hippocampus. Left hippocampal activation significantly mediated the effect of childhood trauma on PTSD (95% CI [0.002, 0.16]), depression (95% CI [0.004, −0.13]), and resilience (95% CI [−0.11, −0.01]) in the Val/Val genotype, but not in the Met carrier group.

## Discussion

Childhood trauma has frequently been associated with development of psychiatric disorders; however, it is necessary to understand the underlying biological mechanisms in order to prevent or limit negative psychiatric outcomes. In this fMRI study, a response-inhibition task was used to examine neural mechanisms by which childhood trauma and *COMT* genotype interact to alter psychiatric risk or resilience. We demonstrated that childhood trauma and the presence of the Met allele was associated with decreased inhibition-related hippocampal activation, whereas childhood trauma in the Val/Val group was related to increased activation in the hippocampus. Second, we investigated the clinical significance of response inhibition-related activation and found that decreased hippocampal activation was associated with more PTSD and depression symptoms. Notably, hippocampal activation also correlated positively with trait resilience. Finally, we showed that in the Val/Val group the effect of childhood trauma on psychiatric risk or resilience was mediated by left hippocampal activation. These data suggest that inhibition-related hippocampal activation may be a mechanism by which childhood trauma and genotype interact to increase risk for trauma-related psychopathology on the one hand and improve resilience on the other hand.

The hippocampus is important for contextual learning and memory, and healthy individuals may recruit the hippocampus to form an association between events or stimuli that are not statistically linked in the environment (52). Therefore, even though we did not directly manipulate contextual cues in this task, hippocampal recruitment during response inhibition likely improves the processing and memorization of information related to the NoGo trials (signaled by the red box that appeared on the screen), which is needed to appropriately guide behavioral responding in the future. In order to successfully cope with early trauma, increased hippocampal activation might help effective behavioral and emotional regulation in later life. We demonstrated that hippocampal activation in subjects with a history of childhood trauma was dependent on the *COMT* gene variation. Childhood trauma load in Val/Val carriers was associated with increased inhibition-related hippocampal activation, and hippocampal activation mediated the relationship between childhood trauma and resilience. This suggests that Val/Val subjects may develop a mechanism to cope with the high levels of early stress by relying more on hippocampal (contextual) information to regulate behavior.

Met carriers, on the other hand, showed reduced hippocampal activation with increasing childhood trauma load. Reduced hippocampal recruitment during response inhibition could indicate a decreased ability to learn from contextual cues, decreased dynamic working memory responses, or decreased memories of salient stimuli, which might set one at risk for psychopathology. Accordingly, we observed that less inhibition-related hippocampal activation was associated with more PTSD and depression symptoms; however, the relationship between childhood trauma and depression (and at trend levels with PTSD) was not mediated by hippocampal activation. The hippocampus is one of the most plastic areas of the brain (53), and could, therefore, be a potential target for early interventions that aim to increase hippocampal-dependent contextual learning and inhibition. Interventions could include certain forms of psychotherapy, inhibition-focused cognitive training, or pharmacological regulation of DA. In a prospective emergency department study where participants were enrolled within 24 h of their traumatic event and scanned at 1 month, the same fMRI task was used, and we observed that more inhibition-related hippocampal activation at 1 month predicted PTSD symptoms at 3 and 6 months (van Rooij, unpublished data). Other studies have also already demonstrated decreased contextual learning and memory in patients suffering from PTSD (54, 55) and major depression (56). Moreover, both disorders (57–60) as well as childhood trauma (61) have been associated with impaired hippocampal functioning. This study is the first that we are aware of to identify GxE factors that impact hippocampal functioning and its relation to psychiatric risk or resilience.

The Val and Met variants of the *COMT* gene have been shown to differ in their DA levels, because the Met substitution decreases enzymatic action of COMT, resulting in less DA breakdown in the synapse and higher extracellular DA levels (4). Exposure to stressful events increases DA activity (62), potentially disturbing the DA balance. It is therefore likely that early-life stress differentially impacts Val/Val versus Met carriers, particularly in those brain regions where COMT is robustly expressed, i.e., hippocampal and prefrontal regions. A balance in DA tone in the PFC and limbic structures is thought to be required for regulating behavior by determining the relevance of external cues and adapting behavior accordingly (63). This might explain why the impact of childhood trauma on inhibition-related hippocampal functioning depends on the *COMT* Val158Met variation. The Met carriers have decreased enzymatic action of COMT and higher extracellular levels of DA. Early stress may therefore have a more disturbing effect on hippocampal-mediated regulated behavior in these individuals compared to the Val/Val individuals. Preclinical studies investigating DA sensitization in adult life concluded that early-life stress, and not stress during adulthood, may establish differential DA sensitivity in the mesolimbic system [reviewed in Ref. (63, 64)]. Notably, all the women in the current study had experienced adult trauma exposure, and including adult trauma in the analyses did not affect the results, supporting the conclusion that specifically childhood trauma interacts with *COMT* genotype to influence hippocampal activation. Also, the absence of genotype effects on hippocampal volume shows that, even though decreased hippocampal volume has been associated with other genetic variants by childhood trauma interactions (65, 66), *COMT* genotype by childhood trauma interaction does not show a significant effect on hippocampal structure, but only function at the level of statistical power available in the current study.

No effect of *COMT* genotype or childhood trauma on response inhibition-related vmPFC activation was demonstrated. This was unexpected given that reduced vmPFC activation during inhibition has been observed previously in PTSD (15), and childhood trauma was related to reduced vmPFC activation in PTSD patients with high levels of childhood trauma (35). Notably, we did not separate PTSD and trauma controls in our analyses. Furthermore, in contrast to our findings, previous studies investigating the effects of *COMT* genotype showed a positive correlation between number of Met alleles and activation in the PFC (67–69) and hippocampus (67, 68). Importantly, these studies used tasks with negative emotional components. When positive stimuli were presented, no increase in other limbic and prefrontal activation (69) or a difference in hippocampal activation (67) was observed. It is therefore important to note that our response-inhibition task involved neutral stimuli. It has been proposed that increased extracellular DA levels in Met carriers may result in enhanced neural sensitivity to negative cues (69). In line with this, several behavioral studies reported an association between mood and anxiety disorders with the Met allele (70–73). In contrast, the Val/Val genotype has been associated with increased risk for schizophrenia (74, 75). This suggests that psychiatric risk is not defined by a *COMT* genotype variant *per se*, but that interactions with environmental factors, such as childhood trauma, are crucial in determining who may be at risk for which disorder.

The whole brain analyses revealed an interaction effect of childhood trauma and *COMT* genotype in the posterior cingulate cortex (PCC). The PCC is involved in internally directed cognition and controlling focus of attention (76). It has previously been associated with *COMT* genotype, as Val/Val demonstrated more PCC activation than Met carriers during a Go/NoGo task (77). In addition, a significant interaction was observed in the paracentral lobe, presupplementary motor area (preSMA), and postcentral gyrus areas that have previously been linked to successful response inhibition (78). Moreover, individual differences in inhibition performance have been related to DA release in (among other regions) the preSMA (79). Given the large effects of the *COMT* Val–Met polymorphism on DA levels and potential downstream alterations in DA receptor availability (80), genotype effects and the interaction with childhood trauma in this region may also make important contributions to risk for trauma-related pathology. Further research is warranted.

A limitation of the current study is the use of retrospective and self-report measures of childhood trauma. We note that the CTQ has been well validated against other measures of childhood trauma (38) and that subjects in our genetic cohorts were not different overall in CTQ levels. Prospective studies following children into adolescence or early adulthood would help to better understand interaction effects of childhood trauma and genetic factors on psychiatric risk. Second, PTSD symptom scores were based on the PSS for consistency with our previous neuroimaging studies (15, 35, 81). Moreover, because the levels of trauma within our sample are very high, the PSS was used to account for PTSD symptoms related to multiple traumas rather than selecting one or more specific events as is usually done in diagnostic interviews. Finally, PTSD symptoms were verified for a subset of participants (66%) using the clinician-administered PTSD scale (CAPS). PTSD symptom scores using CAPS and PSS were positively correlated (*r* = 0.58, *p* < 0.001). Third, we used FreeSurfer for volumetric measures instead of manual tracing. As this is an automated method, there are some concerns about its consistency with manual tracing. Yet, recent studies showed that estimated hippocampal volumes with FreeSurfer were highly correlated with manually traced volumes (82), and FreeSurfer was shown to have better reproducibility than manual tracing (83). Furthermore, in our sample only one individual was homozygous for the Met/Met genotype. Therefore, we could only compare Met carriers to the Val/Val genotype. Larger samples are warranted to compare the three different genotypes and investigate if homozygous Met carriers have an additional disadvantage. Furthermore, replication in a separate cohort is warranted, because of the reduction in usable data due to head motion, anatomical abnormalities, scanner issues, and poor task performance. Also, even though this is an understudied population in psychiatric neuroimaging research, the unique character of this sample may also limit the generalizability of the findings.

Our findings suggest that *COMT* genotype is an important moderator of the impact of early-life stress on hippocampal recruitment during inhibition, which, in turn, relates to psychiatric risk or resilience. Hippocampal recruitment may improve the ability to use information from the changing environment to guide behavior and may thereby enhance resilience in Val/Val individuals who were exposed to trauma early in life. On the other hand, in Met carriers early trauma was negatively associated with hippocampal function. Because the hippocampus is highly plastic, it represents an excellent potential target for early interventions in Met carriers to enhance hippocampal-dependent contextual learning and inhibition. Future research on the effectiveness of targeted interventions that increase resilience would be of great interest, particularly given the high rate of psychiatric disorders and difficulty of treating these disorders in survivors of childhood trauma.

## Author Contributions

SR, JS, TE, NF, and TJ performed functional and structural MRI data collection and analyses. AS, KK, AL, and KR performed the genetic analyses. KR and TJ obtained funding for this study. SR, JS, and TJ wrote the paper and all authors read, commented on, and approved the final version.

## Conflict of Interest Statement

The authors declare that the research was conducted in the absence of any commercial or financial relationships that could be construed as a potential conflict of interest.

## Footnotes

^1^http://www.fil.ion.ucl.ac.uk/spm

^2^http://dbm.neuro.uni-jena.de/vbm
