# Supplementary material for: Childhood Trauma and COMT Genotype Interact to Increase Hippocampal Activation in Resilient Individuals
Source: Front Psychiatry. 2016 Sep 14;7:156. doi: 10.3389/fpsyt.2016.00156 (PMC5021680; doi:10.3389/fpsyt.2016.00156)
Supplement: Supplementary file 1 [file Table_1.DOCX]

**Supplementary Table S1**

**Results of moderated regression analyses, correcting for ancestry**

| a. Left hippocampus | |  |  |
| --- | --- | --- | --- |
| **Standardized B** | | **t** | **p-value** |
| Age | -0.061 | -0.520 | 0.605 |
| PC1 | -0.048 | -0.393 | 0.695 |
| CTQ | -0.014 | -0.116 | 0.908 |
| *COMT* | -0.278 | -2.302 | 0.025* |
| CTQ**COMT* | -0.284 | -2.478 | 0.016* |
|  |  |  |  |
| b. Right hippocampus | |  |  |
| **Standardized B** | | **t** | **p-value** |
| Age | -0.070 | -0.580 | 0.570 |
| PC1 | 0.018 | 0.143 | 0.887 |
| CTQ | -0.021 | -0.176 | 0.861 |
| *COMT* | -0.202 | -1.628 | 0.108 |
| CTQ**COMT* | -0.244 | -2.069 | 0.043* |
|  |  |  |  |
| c. vmPFC |  |  |  |
| **Standardized B** | | **t** | **p-value** |
| Age | 0.042 | 0.326 | 0.745 |
| PC1 | -0.109 | -0.834 | 0.408 |
| CTQ | -0.017 | -0.131 | 0.896 |
| *COMT* | 0.009 | 0.067 | 0.947 |
| CTQ**COMT* | 0.060 | 0.482 | 0.631 |

CTQ, Childhood Trauma Questionnaire(37, 38); PC1, Principal Component 1
