# Supplementary material for: Childhood Trauma and COMT Genotype Interact to Increase Hippocampal Activation in Resilient Individuals
Source: Front Psychiatry. 2016 Sep 14;7:156. doi: 10.3389/fpsyt.2016.00156 (PMC5021680; doi:10.3389/fpsyt.2016.00156)
Supplement: Supplementary file 2 [file Table_2.DOCX]

**S3 Supplementary Table 2**

**Results of moderated regression analyses, correcting for ancestry and adult trauma**

| a. Left hippocampus | |  |  |
| --- | --- | --- | --- |
| **Standardized B** | | **t** | **p-value** |
| Age | -0.052 | -0.444 | 0.659 |
| PC1 | -0.047 | -0.388 | 0.699 |
| TEI | -0.123 | -1.046 | 0.300 |
| CTQ | 0.009 | 0.075 | 0.940 |
| *COMT* | -0.273 | -2.257 | 0.027* |
| CTQ**COMT* | -0.277 | -2.410 | 0.019* |
|  |  |  |  |
| b. Right hippocampus | |  |  |
| **Standardized B** | | **t** | **p-value** |
| Age | -0.061 | -0.505 | 0.615 |
| PC1 | 0.018 | 0.148 | 0.883 |
| TEI | -0.117 | -0.968 | 0.337 |
| CTQ | 0.000 | 0.002 | 0.999 |
| *COMT* | -0.197 | -1.585 | 0.118 |
| CTQ**COMT* | -0.237 | -2.004 | 0.049* |
|  |  |  |  |
| c. vmPFC |  |  |  |
| **Standardized B** | | **t** | **p-value** |
| Age | 0.032 | 0.252 | 0.802 |
| PC1 | -0.110 | -0.839 | 0.404 |
| TEI | 0.129 | 1.018 | 0.313 |
| CTQ | -0.040 | -0.313 | 0.755 |
| *COMT* | 0.003 | 0.024 | 0.981 |
| CTQ**COMT* | 0.052 | 0.418 | 0.677 |

CTQ, Childhood Trauma Questionnaire(37, 38); PC1, Principal Component 1; TEI, Traumatic Events Inventory(39).
