# Supplementary material for: Childhood Trauma and COMT Genotype Interact to Increase Hippocampal Activation in Resilient Individuals
Source: Front Psychiatry. 2016 Sep 14;7:156. doi: 10.3389/fpsyt.2016.00156 (PMC5021680; doi:10.3389/fpsyt.2016.00156)
Supplement: Supplementary file 3 [file Table_3.DOCX]

**S4 Supplementary Table 3**

**Correlation analyses with age, CTQ and PC1 as covariates**

|  |  | **PTSD severity** | | **Depression severity** | | **Resilience** | |
| --- | --- | --- | --- | --- | --- | --- | --- |
|  | Covariates | ***r*** | ***p*** | ***r*** | ***p*** | ***r*** | ***p*** |
| **Left Hippocampus** | None | -0.27 | 0.023* | -0.29 | 0.011* | 0.34 | 0.007* |
|  | Age | -0.29 | 0.015* | -0.31 | 0.008* | 0.36 | 0.004* |
|  | CTQ total | -0.25 | 0.033* | -0.28 | 0.018* | 0.32 | 0.011* |
|  | PC1 | -0.26 | 0.031* | -0.29 | 0.016* | 0.35 | 0.008* |
|  | Age, CTQ, and PC1 | -0.27 | 0.026* | -0.29 | 0.016* | 0.35 | 0.009* |
|  |  |  |  |  |  |  |  |
| **Right Hippocampus** | None | -0.21 | 0.074 | -0.21 | 0.079 | 0.29 | 0.022* |
|  | Age | -0.24 | 0.047* | -0.23 | 0.057 | 0.33 | 0.010* |
|  | CTQ total | -0.20 | 0.092 | -0.19 | 0.103 | 0.28 | 0.029* |
|  | PC1 | -0.21 | 0.086 | -0.21 | 0.082 | 0.29 | 0.025* |
|  | Age, CTQ, and PC1 | -0.22 | 0.075 | -0.21 | 0.086 | 0.30 | 0.023* |

CTQ, Childhood Trauma Questionnaire(37, 38); PC1, Principal Component 1
