# Supplementary material for: Childhood Trauma and COMT Genotype Interact to Increase Hippocampal Activation in Resilient Individuals
Source: Front Psychiatry. 2016 Sep 14;7:156. doi: 10.3389/fpsyt.2016.00156 (PMC5021680; doi:10.3389/fpsyt.2016.00156)
Supplement: Supplementary file 4 [file Image_1.PDF]

Supplemental Figure S1

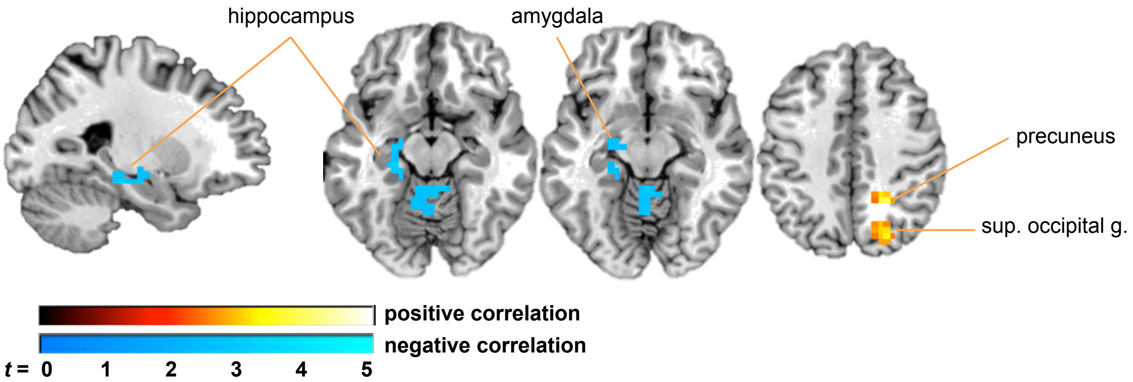

Figure 1 Whole brain activation during the response inhibition task showing a positive or negative correlation with number of Met alleles. A significant main effect of *COMT* genotype was observed in the hippocampus and amygdala (more activation in Met carriers), and precuneus and superior occipital gyrus (more activation in Val/Val subjects).
